# Addressing hospitalisations with non-error-free data by generalised SEIR modelling of COVID-19 pandemic

**DOI:** 10.1038/s41598-021-98975-w

**Published:** 2021-10-04

**Authors:** Jorge M. Mendes, Pedro S. Coelho

**Affiliations:** NOVAIMS, Universidade Nova de Lisboa, Campus de Campolide, 1070-312 Lisbon, Portugal

**Keywords:** Viral infection, Statistics

## Abstract

Successive generalisations of the basic SEIR model have been proposed to accommodate the different needs of the organisations handling the SARS-CoV-2 epidemic. These generalisations have not been able until today to represent the potential of the epidemic to overwhelm hospital capacity until today. This work builds on previous generalisations, including a new compartment for hospital occupancy that allows accounting for the infected patients that need specialised medical attention. Consequently, a deeper understanding of the hospitalisations rate and probability as well as of the recovery rates for hospitalised and non-hospitalised individuals is achieved, offering new information and predictions of crucial importance for the planning of the health systems and global epidemic response. Additionally, a new methodology to calibrate epidemic flows between compartments is proposed. We conclude that the two-step calibration procedure is able to recalibrate non-error-free data and showed crucial to reconstruct the series in a specific situation characterised by significant errors over the official recovery cases. The performed modelling also allowed us to understand how effective the several interventions (lockdown or other mobility restriction measures) were, offering insight for helping public authorities to set the timing and intensity of the measures in order to avoid the implosion of the health systems.

## Introduction

In December 2019, a new coronavirus named severe acute respiratory syndrome-coronavirus-2 (SARS-CoV-2), causing severe acute respiratory disease emerged in the region of Wuhan, China^1,2^. SARS-CoV-2 is an acute respiratory infectious disease that spreads through the respiratory tract by droplets, respiratory secretions, and person-to-person contact^3,4^.

**Figure 1:**
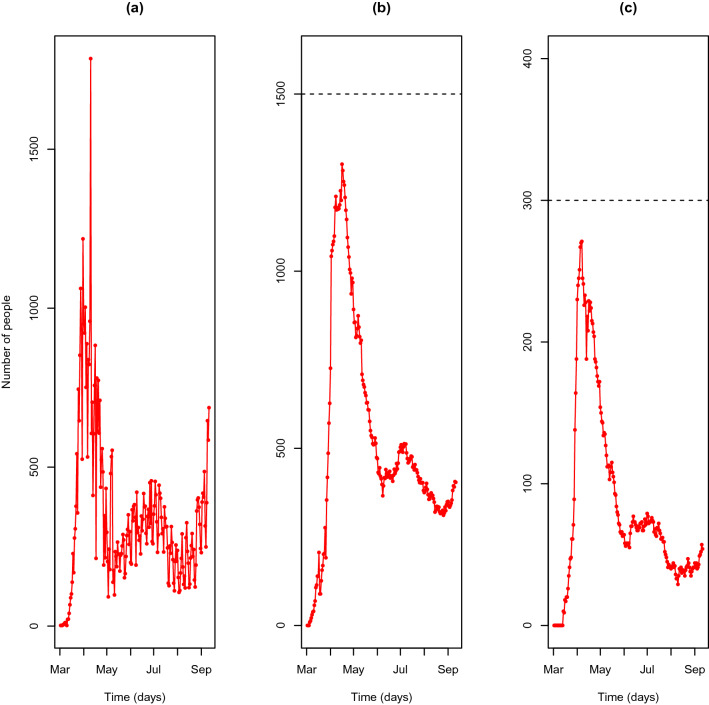
Course of the COVID-19 epidemic in Portugal. (**a**) Daily incidence of COVID-19 cases in Portugal, (**b**) Daily hospital occupancy due to COVID-19 in Portugal, (**c**) Daily ICU occupancy due to COVID-19 in Portugal. Data collected from the daily bulletins issued by *Direção-Geral de Saúde* (Directorate-General of Health, DGS), the Portuguese health authority. The dashed horizontal lines show the generally accepted national health system limits.

At the time of the writing of this introduction, a “second wave” of new COVID-19 infections is striking some European countries, while in South America and North America a first wave is spreading at an alarming rate. Its transmissibility is high (reproductive number, $R_0$, estimated to be between 2 and 3^5–8^), indicating that a large proportion of the world population can be infected. As a significant number of patients have severe symptoms and need dedicated medical care (around one in every five people who are infected), the potential to overwhelm our health care systems and intensive care units is a real threat^9^. In Portugal, after an incidence spike in early April (cf. Fig. 1a), the lockdown measures had been easing in early May, resulting in an increase of the daily incidence of new cases (cf. Fig. 1a). Although the Portuguese national health system limits (dashed horizontal lines in Fig. 1b,c) have never been reached, the current course of the epidemic raises critical management issues, in particular, because the more significant growth of the second wave is threatening to overcome hospitalisation capacity in the short term.

Much work has been done so far on the COVID-19 outbreak course using the so-called compartmental models^10–12^. The origin of compartmental models dates back to the early 20th century with the seminal work of Kermack and McKendrick (in 1927)^13^. Compartmental models simplify the mathematical modelling of infectious diseases. The population is assigned to compartments and may progress between compartments. Compartmental order usually shows the flow patterns between the compartments; for example, SIS means susceptible, infectious, and then susceptible again^14^. The models are most often run with ordinary differential equations (which are deterministic), but can also be used with a stochastic framework, as it is the case here^14^.

Compartmental models allow predicting how a disease spreads out through the total number infected, the duration of an epidemic, and infection reproductive number ($R_0$), among other important epidemic course parameters. Moreover, such models can show how different public health interventions (e.g. vaccination, limited social contacts, lockdown) may affect the outcome of the epidemic.

The basic compartmental model is the SIR model, and other more complex variations are derivatives of the basic SIR model^13^. The SIR model comprises three compartments: *S* for the number of susceptible, *I* for the number of infectious, and *R* for the number of removed (recovered, deceased, or immune) individuals at a particular time. This model assumes incidence grows exponentially, which is not in agreement with the observed epidemic course, as the measures adopted by public health authorities at different moments, as well as the change of human behaviours tend to flatten the parabolic incidence curve. Therefore, its predictive value for infectious diseases that are transmitted from humans to humans, without any change on the constant transmission rate is of limited usefulness.

To represent that the number of susceptible, infectious and removed individuals may vary over time (even if the total population size remains constant) a time index, *t*, might be added: $S_t$, $I_t$ and $R_t$. For a specific disease within a particular population, these functions may be worked out to predict possible outbreaks and bring them under control.

For infections with the characteristics of SARS-CoV-2, there is a significant incubation period during which individuals have been infected but are not yet infectious themselves^9,15–19^. During this period the individual is in compartment *E* (for exposed), resulting in the so-called SEIR model. The SEIR model’s basic version is still short for our current needs, especially regarding the burden put in the health system. Indeed, the basic version of the SEIR model is unable to distinguish between deceased and recovered people and provide any helpful information on the daily burden put on a health system by individuals in need of medical care. Therefore successive generalisations of the basic SEIR model have been proposed to accommodate either the different needs of the different organisations handling epidemic-related problems or the need to account for public health data not specifically used in the basic SEIR model. This work seeks to pursue these needs and extend such generalisations. For public health officers, the number of confirmed cases is the part of the iceberg that is visible as many more people are infected but still not infectious because the virus is still in the replication phase (incubation period). Indeed, these people would come up as confirmed cases a few days ahead. Knowing in advance how the number of confirmed cases might evolve is of crucial importance for planning reasons.

Moreover, it is of unquestionable value for health system officers to know the share of confirmed COVID-19 confirmed cases who are going to need for specialised medical care and how many of them will recover or die. Additionally, for researchers with no access to micro-data files (due to unavailability or lack of data quality) it is impossible to know some of the flows between some compartments of the SEIR model’s generalisations. This work also addresses this problem, establishing a methodology to calibrate epidemic flows between compartments, that is relevant to recalibrate non-error-free data. To accomplish these goals, we build on the work of^20^ and decided to propose several extensions and restrictions, including the consideration of a new compartment representing hospital occupancy.

This work is organised as follows: “The SEIQRHD model” section describes the classic SEIR model summarily and its proposed generalisation to meet our aims. It also des-cribes our proposals for accommodating some of the characteristics already mentioned as well as others suggested by the empirical analysis of the Portuguese epidemic course; “Data and empirical analysis” section describes the used data and the major data processing tasks prior to modelling using a Bayesian framework and, finaly, “Bayesian hierarchical modelling” section presents the hierarchial Bayesian framework used for parameters estimation. “Results” and “Discussion” sections respectively present and discuss, respectively, the achieved results for the case of Portuguese epidemic course and the main implications for managing and controlling the course of the outbreak.

## Data and model

### The SEIQRHD model

**Figure 2:**
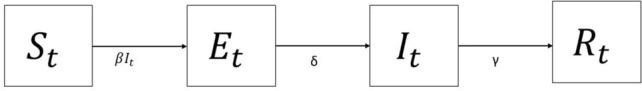
Basic SEIR model.

Assuming the vital dynamics (birth, deaths and migration) may be neglected (which is not a hard assumption given the short period of the pandemic) the basic SEIR model is defined as in Fig. 2.

The boxes represent the compartments (or states) and arrows represent transitions from one compartment to another. $S_t$ is the number of people susceptible at time *t*, $E_t$ is the number of people exposed to the infection at time *t* (people that become infected but not infective), $I_t$ is the number of infective people infected at time *t* and $R_t$: number of people removed (recovered or deceased) at time *t*. The parameter $\beta$ denotes the transmission rate (the expected amount of people an infected person infects per day and is the result of the contact rate—the number of people an average person enters into contact with each day—and the probability that a contact provokes the transmission of the disease). It is multiplied by the ratio *S*/*N* to avoid counting contacts between two people who cannot infect each other (e.g., because one of them has already recovered and immune, or because both are infective^21^). The parameter $\delta$ is the disease incubation rate (the inverse of the incubation period in days), and $\gamma$ is the removal rate (the inverse of the removal period in days). When some of these three parameters are considered fixed, they do not, in general, fit the observed course of the current epidemic. For example, the social distancing measures or even the lockdown damp the transmission rate down by reducing the daily contacts one person regularly has; or the improvement and gained experience of the health care system pull the removal rate up by lowering the recovery period or the death rate. Making these two parameters, $\beta _t$ and $\gamma _t$, functions of time, with user-defined functional forms, is the solution to describe the course of the disease spread appropriately. As already mentioned, for epidemics that last for a short period (some months, for instance) it does not worth to account for demographics dynamics (births, migration, deaths by other causes) as the population can be considered fixed. Nonetheless, for the cases whenever the demographics may impact other transitions might also be considered also to account for those dynamics.

Based on the SEIR model, many measures of the epidemic development measures might be assessed^15,21–24^. We refer to^25^ as an excellent review of the recent work done in this area. The SEIR model was also used to compare the effects of the Hubei province’s lockdown on the transmission dynamics in Wuhan and Beijing^16^.

As the SEIR model can generate interpretable results, wave derivatives are being developed^25^ (a) to simulate the processes of transmission from infection source; (b) to assess the transmission risk and prediction of patient number based into two subpopulations of infected people, those the quarantined and unquarantined; (c) to simulate the incubation period and the period before recovery.

**Figure 3:**
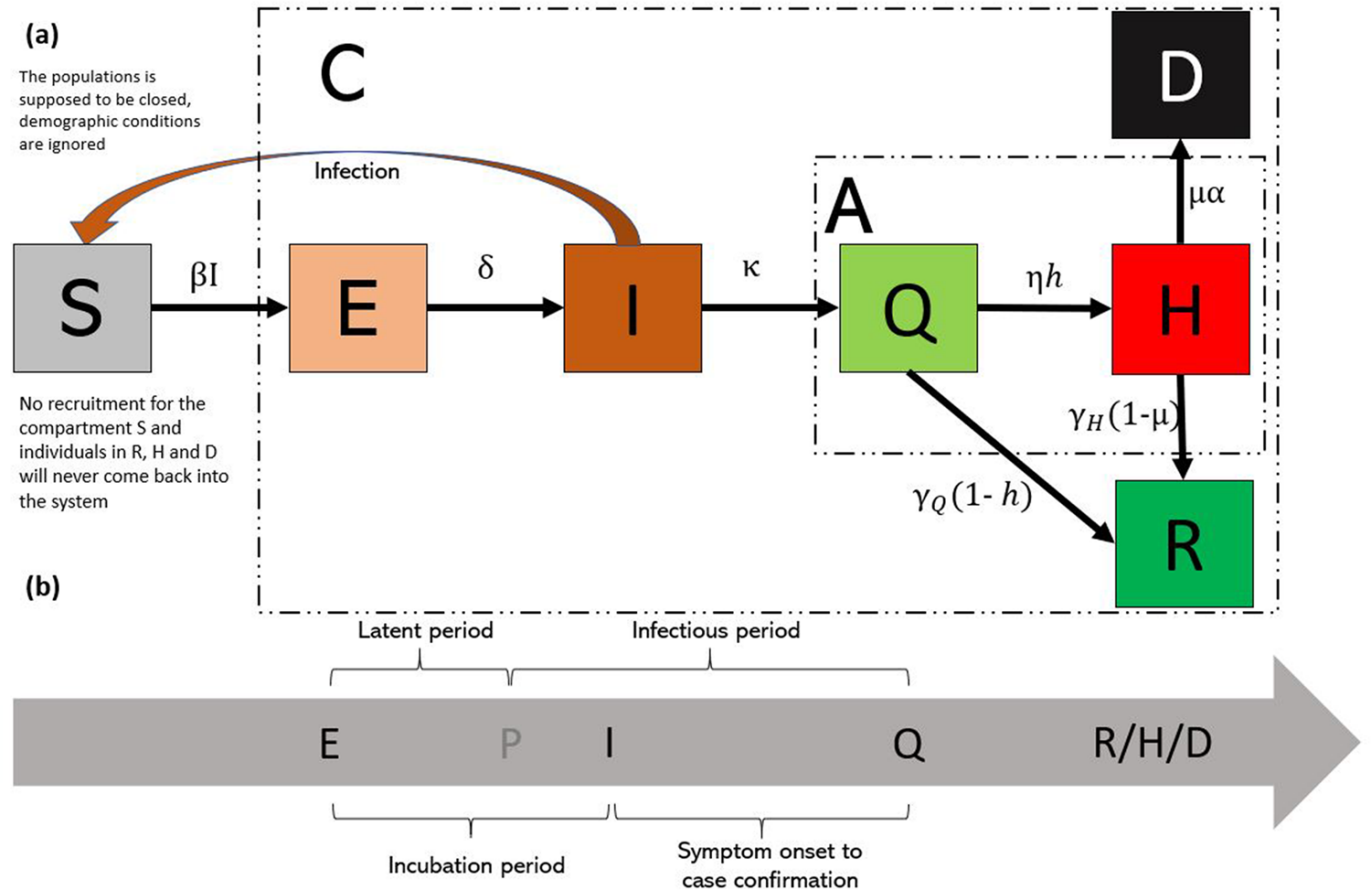
The SEIQRHD model used in this work. (**a**) SEIQRHD model. (**b**) Schematic representation of temporal evolution between compartments E, I and Q.

Many of these works consider different generalisations of the basic SEIR model as those four compartments are often insufficient to describe a much more complex reality. First, considering only one compartment to account for removed people, *R*, is not enough to describe the evolution of patients that recover from the infection and the share that deceases. Secondly, the compartment *I* does not match the observed amount of the infected people in many situations as many infected people are asymptomatic or only experience mild symptoms (and they are not accounted for by the figures released out by the health authorities) or it does not account for the isolation (quarantine) usually imposed by health authorities making these people no longer infectious. Indeed, the daily number of “confirmed infections” corresponds to people who are isolated (quarantined) to avoid new infection chains. As a solution, many authors^20,25^ have been proposing generalisations of this basic SEIR model by splitting the compartment, *R* into two compartments, one to account for the infected people that genuinely recover from the disease, *R*, and a second one to account for the mortality induced by the infection, *D*. Moreover, to precisely match the observed number of confirmed cases, one additional compartment, *Q*, has been considered immediately after the *I* compartment^20^. Building on the work of^25^ we propose an additional compartment to account for hospital occupancy, *H*. Figure 3a reproduces the generalisation of the SEIR model used in this work. Figure 3b describes schematically the temporal evolution between compartments *E*, *I* and *Q*.

Figure 3a contains two additional compartments to keep track of the cumulative number of COVID-19 infections, *C*, and the current number of ill individuals, the so-called “active cases”, *A*. This models includes other not yet described parameters. Further ahead, we elaborate on the potential functional forms to describe their temporal evolution and use the opportunity to define them.

The following differential equations describe this is generalisation of the SEIR model (SEIQRHD):

1 $$\begin{aligned} \begin{array}{llll} \partial S_t/\partial t &{} = &{} -\beta S_t \displaystyle \frac{I_t}{N}\\ \partial E_t/\partial t &{} = &{} \beta S_t \displaystyle \frac{I_t}{N} - \delta E_t\\ \partial I_t/\partial t &{} = &{} \delta E_t - \kappa I_t\\ \partial Q_t/\partial t &{} = &{} \kappa I_t - \eta h Q_t - \gamma _{Q_t} (1-h) Q_t\\ \partial H_t/\partial t &{} = &{} \eta h Q_t - \gamma _H (1-\mu _t) H_t - \mu \alpha H_t\\ \partial R_t/\partial t &{} = &{} \gamma _{Q_t} (1-h) Q_t + \gamma _H (1-\mu ) H_t\\ \partial D_t/\partial t &{} = &{} \mu \alpha H_t\\ \partial C_t/\partial t &{} = &{} \delta E_t\\ \partial A_t/\partial t &{} = &{} \kappa I_t - \gamma _{Q_t} (1-h) Q_t - \gamma _H (1-\mu ) H_t - \mu \alpha H_t\\ N &{} = &{} S_t + E_t + I_t + Q_t + R_t + H_t + D_t \end{array} \end{aligned}$$

The discussion on whether the parameters are fixed is done further ahead; therefore, the subscript *t* in the parameters is omitted for the time being. Some of the model coefficients might be considered fixed ($\delta$, $\kappa$, $\alpha$), but others ($\beta$, $\eta$, *h*, $\gamma$ and $\mu$) vary in time according to some specific functional form.

The parameter $\delta$ is the inverse of the average incubation period and governs the lag between having undergone an infectious contact and showing symptoms. It is generally considered fixed because it depends mainly on the the infectious agent’s characteristics.

The parameter $\kappa$ is the inverse of the average period taken to isolate a symptomatic person, which usually happens after the person has been tested positive. Therefore, the quarantined compartment matches the “active confirmed cases” in many official data sources in most of the developed countries and the quarantined individuals are excluded from the infectious compartment (*I*) because they are supposed to be isolated. The time required to isolate a person with symptoms depends mainly on the protocol followed by the health authorities which with some minor adjustments is constant. As explained further ahead, we consider this parameter fixed after an initial period of adaptation, and use an empirical estimate, jointly with a literature-based value of $\delta$ to estimated the flows between compartment *S* and *E* and *E* and *I*. Within the hierarchical Bayesian model they are even considered fixed.

As the lockdown and social distancing measures impact on the number of individuals a person comes in contact with on a daily basis, the parameter $\beta$ should not be considered constant.

The parameters $\gamma _Q$ and $\gamma _H$ are the recovery rate for hospitalised and quarantined people. They correspond to the inverse of the average time required for an active case (quarantined/hospitalised) to be classified as recovered. They provide precious information about how fast the people may recover from the disease (in days, in general). We believe that both $\gamma _Q$ and $\gamma _H$ are related to how a health system can improve its capability to treat people over time (e.g., with the introduction of a new therapeutic strategies). However, $\gamma _Q$ is much more dependent on recovery confirmation protocols and therefore, much more susceptible to increase over time.

The outer flows from compartment *Q* depend on $\gamma _Q$, already discussed, $\eta$ and *h*. The rate at which quarantined individuals are hospitalised, $\eta$ ($\eta ^-1$ is the average period spent in compartment *Q*), mainly depends on the illness severity and health system capacity to accept ill individuals, whilst the share of people in need of specialised medical care, *h*, mainly depends on the population health conditions, as well as the mix of the quarantine compartments regarding demographic characteristics as gender and age. Whilst the former might be considered fixed to some extent, the latter, due to the mentioned reasons cannot be. Indeed, recent empirical analysis shows that after the epidemic spike in late April 2020, the share of COVID-19 patients admitted to a hospital decreased from almost 100%, in the early days of the epidemic, to a tiny constant value, in late July. This phenomenon might be explained by the uncertainty and lack of knowledge of the disease course in the early days of the epidemic. However, as time goes by, the medical practitioners learn and become more selective about the patients to be admitted to a hospital. Moreover, as the prevalence curve increases, the health systems tend to avoid the intolerable burden and become more demanding on the hospitalisation requirements. Therefore, there is enough evidence to consider $\eta$ a constant parameter and $h_t$ a time-varying parameter.

The parameter $\mu$ is the fatality rate and provides information on the proportion of hospitalised individuals who, unfortunately, die. It depends either on the resilience of the patients and the severity of the disease. As this variation is not time-related we consider it to be fixed. It is worth mentioning here that any transition from the quarantined *Q* to the death *D* compartments is not considered at all, as any ill and quarantined individual that gets worse is, at some point in time, admitted to a hospital and eventually recovers or dies. The parameter $\alpha$ denotes the transition rate between *H* and *D*. Its inverse corresponds to the time a hospitalised individual takes to die. We anticipate these parameters to be highly variable as, in the ultimate analysis, it depends on the individuals themselves, more than the quality of the health care provided, but we do not see any reason to consider it a time-varying parameter.

Many infected people experience mild or no symptoms at all and are not accounted for (ascertained). Therefore, the Infection Fatality Rate (IFR), i.e. the number of deaths as a proportion of all persons infected with the SARS-CoV-2 novel coronavirus is expected to be much lower than the Case Fatality Rate (CFR), the proportion among confirmed cases. Some works have reported values for the ascertainment rate between 0.4 and 14% in Wuhan^7,26–30^, 28.4% in Italy^16^ and just 0.23% in Iran^31^. Unfortunately, the available data does not allow the estimation of this ascertained rate^24^, that account for an increased number of people in each compartment than the actual figures.

For modelling and empirical data analysis performed hereafter one uses a discrete-time approximation to the stochastic continuous-time SEIQRHD model defined in Eq. (1). Consider a time interval $(t, t + h)$, where *h* represents the length between the time points at which measurements are taken, here $h = 1$ day. Let $dSE_t$ denote the daily number of susceptible individuals who become infected, $dSI_t$ the number of cases by date of symptom onset, $dIQ_t$ the daily number of confirmed cases, $dQR_t$ the daily number of quarantined cases who recover, $dHR_t$ the daily number of hospitalised cases who recover, $dQH_t$ the daily number of quarantined cases admitted to a hospital for more specialised care and $dHD_t$ the daily number of cases who decease. Given initial conditions $S_0 = s_0$, $E_0 = e_0$, $I_0 = i_0$, $Q_t=q_0$ $R_0 = 0$, $H_t=0$ and $D_0=0$, and the population size *N*, the discretised stochastic SEIQRHD model is specified by:

2 $$\begin{aligned} \begin{array}{llll} S_{t+1} &{} = &{} S_t - dSE_t \\ E_{t+1} &{} = &{} E_t + dSE_t - dEI_t\\ I_{t+1} &{} = &{} I_t + dEI_t - dIQ_t\\ Q_{t+1} &{} = &{} Q_t + dIQ_t - dQH_t - dQR_t\\ H_{t+1} &{} = &{} H_t + dQH_t - dHR_t - dHD_t\\ R_{t+1} &{} = &{} R_t + dQR_t + dHR_t\\ D_{t+1} &{} = &{} D_t + dHD_t\\ C_{t+1} &{} = &{} C_t + dSE_t\\ A_{t+1} &{} = &{} A_t + dIQ_t - dHD_t - dHR_t - dQR_t\\ dSE_t &{} = &{} \beta S_t \displaystyle \frac{I_t}{N} \\ dEI_t &{} = &{} \delta E_t\\ dIQ_t &{} = &{} \kappa I_t\\ dQH_t &{} = &{} \eta h_t Q_t\\ dQR_t &{} = &{} \gamma _Q (1-h_t) Q_t\\ dHR_t &{} = &{} \gamma _H (1 - \mu ) H_t\\ dHD_t &{} = &{} \mu \alpha H_t\\ N &{} = &{} S_t + E_t + I_t + Q_t + R_t + H_t + D_t, \end{array} \end{aligned}$$

This set of equations, jointly with the initial conditions, define the SEIQRHD model used in this work.

### Data and empirical analysis

We collected the epidemic data from the *Direção-Geral de Saúde* (Directorate-General of Health, DGS), the Portuguese health authority. Using the SEIQRHD model described in the previous section we propose to disclose either the course of the Portuguese SARS-CoV-2 epidemic curves at the country level on a daily basis as long as the analysis of the information provided by model parameters. The results of the work based on a simplified version of the model can be found on https://insights.cotec.pt/ on the mosaic *Modelos epidemiológicos*. Data collection and analysis has taken into consideration guidelines of good practices (e.g.^32^).

**Figure 4:**
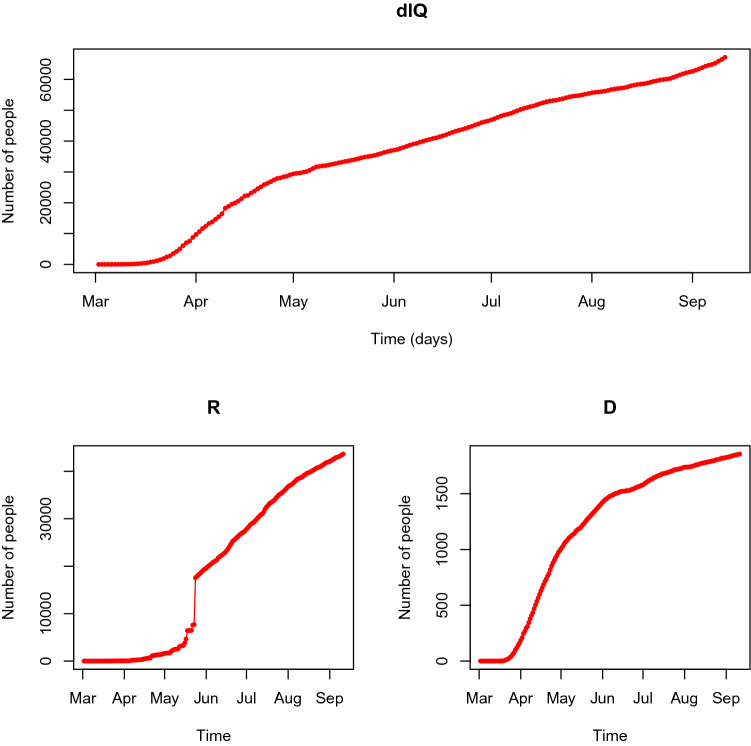
Course of the COVID-19 epidemic in Portugal. (**a**) Confirmed infected cases (cumulative). (**b**) Recovered cases (cumulative). (**c**) Deaths (cumulative).

Since the beginning of the pandemic, DGS releases daily data on its course (https://covid19.min-saude.pt/ponto-de-situacao-atual-em-portugal/). The key released variables are number of confirmed cases, number of deaths (nationwide and by health region), number of recovered cases, hospital occupancy, intensive care unit occupancy (nationwide), as well as the characterisation of the positive cases by origin, gender and age group. The figures released by DGS are used for modelling purposes as explained below. By September 11th, 2020, the course of the epidemic is as Figs. 1 and 4 describe.

The epidemic started on March 2nd, 2020 with the first two cases. Some unexpected spikes of daily recovered cases can be observed between May 14th and 24th 2020. This information corresponds to the release of recovered cases than have recovering during the previous weeks but have not been accounted for before as the recover notifications issued by the primary health care system were not included in the released figures. Moreover, it is globally clear that the released data about recoveries does not reflect the actual daily number of cases as some of these cases tend to be accumulated and only released later on. Therefore, we decided to proceed to a calibration on the number of recovered cases. This calibration impacts the hospital occupancy ($H_t$) and quarantined individuals ($Q_t$) and the two flows of recovered cases coming into the compartment $R_t$. Only the series of daily new quarantined ($dIQ_t$), and the daily states of the active ($A_t=Q_t+H_t$), recovered ($R_t$), hospitalised ($H_t$), and death ($D_t$) cases are released by DGS, but the fact that the number of recoveries is released with some delay, also leads to inaccuracies over the stock of quarantined cases and consequently on the active cases. To account for the mentioned dependencies we decided to calibrate the unknown flows ($dSE_t$, $dEI_t$, $dQH_t$, $dQR_t$, $dHR_t$), using the available data: (a) the daily number of new confirmed cases, $dIQ_t$, (b) the daily number of new deaths, $dHD_t$, and its daily stock $D_t$, (c) the daily number of new recovered cases, $dHR_t+dQR_t$ and is stock $R_t$ (d) the daily number of hospitalisations, $H_t$ and (e) the daily number of quarantined cases $Q_t=A_t-H_t$.

**Table 1:** Mean time and corresponding standard deviation for calibration of distribution of daily incidences of non-observed series.

Estimate	Mean	Std deviation
Incubation period (in days) of SARS-Cov-2	6.50	2.60
Time (in days) from symptom onset to case confirmation (prior April, 2nd)	5.34	4.80
Time (in days) from symptom onset to case confirmation (post April, 2nd)	4.73	6.45

**Figure 5:**
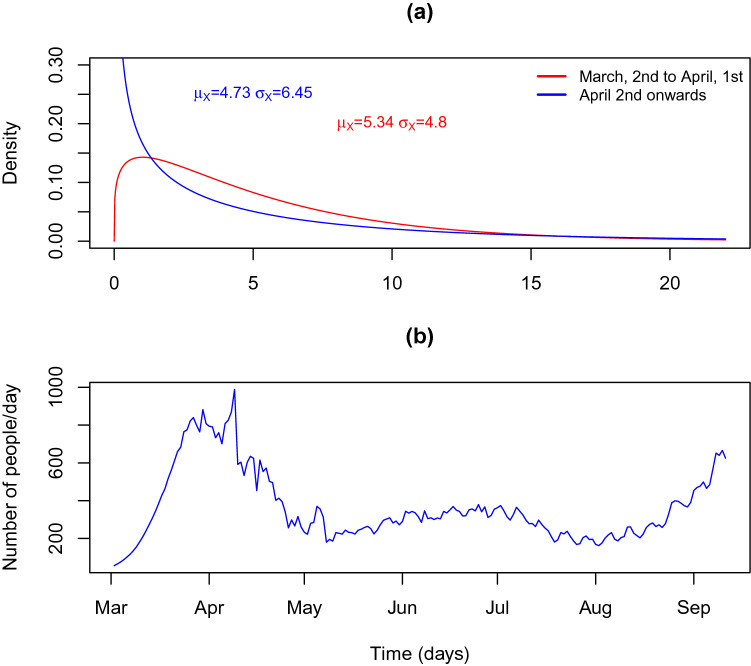
Distribution of time from symptom onset to case confirmation and reporting and the resulting curve of the estimated curve of daily infective people. (**a**) Distribution of time from symptom onset to case confirmation and reporting. (**b**) Estimated infectious ($dEI_t$) cases in Portugal.

**Figure 6:**
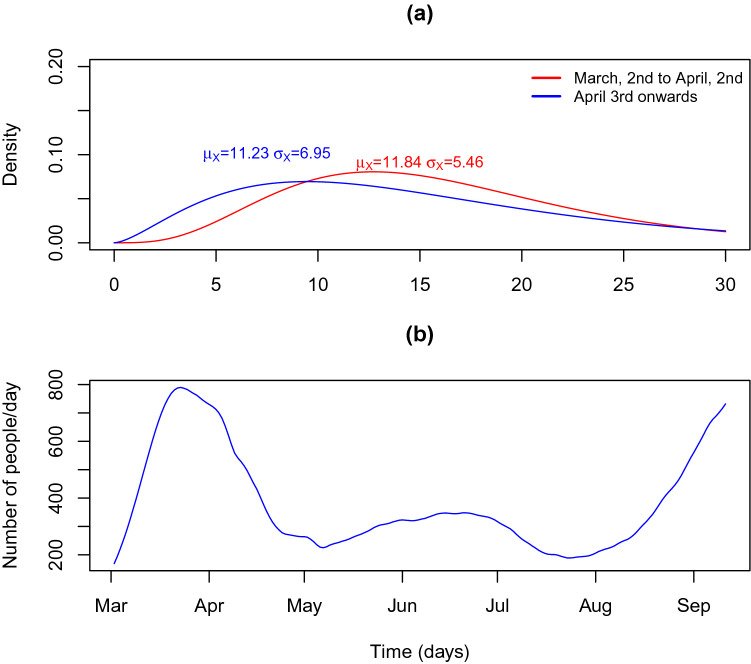
Distribution of time from infection to case confirmation and reporting and resulting curve of estimated curve of daily infective people. (**a**) Distribution of time from infection to case confirmation and reporting. (**b**) Estimated infections ($dSE_t$) in Portugal.

The calibration methodology is a two-step procedure. In the first step we calibrate the series of exposed ($dSE_t$) and infected ($dEI_t$) based on the incubation period of COVID-19 and the time from symptom onset to laboratory confirmation (based on the analysis of COVID-19 micro-data file provided by DGS). Table 1 summarises the distribution parameters used to calibrate the daily incidences of $dSE_t$ and $dEI_t$ as described in Figs. 5 and 6. The median incubation period of SARS-Cov-2 has been estimated at approximately 5–6 days, with a range between 1 and 21 days^9,15–18,33–35^. We used the value provided by^35^ for the gamma distribution. We assume that the mean time from symptom onset to diagnosis confirmation in Portugal decreased from 5.34 days (with a standard deviation of 4.8 days) in the early period of the epidemic (early March) to 4.74 days (standard deviation of 6.5 days) in early April. These values are based on empirical analysis of microdata file provided by DGS. The file provides information for positive cases on the dates of symptom onset and test results. The latter is used to start counting the isolation period. The time necessary to isolate an individual has decreased steeply the in early days of the epidemic to a steady value, achieved one April 2nd, around 1 month after the epidemic started. To reflect the above, we modelled the time between case reporting and symptom onset with a Gamma distribution with $\mu _X/\sigma _X$ parameter of 5.34/4.80 on March 2, decreasing linearly to 4.72/6.45 on April 2 and after (Fig. 5a). Similarly the time between case exposure (infection with SARS-CoV-2) and diagnosis confirmation (based on empirical analysis DGS provided microdata) was modelled with a Gamma distribution with $\mu _X/\sigma _X$ parameter of 11.84/7.4 on March 2, decreasing linearly to 11.23/9.1 on April 2 and after (Fig. 6a).

The calibrated series $dEI_t$ and $dSE_t$ are shown is Figs. 5b and 6b.

In the second step, as the available data provide no information on the daily flows from the *Q* compartment to the *H* and *R* compartments ($dQH_t$, $dQR_t$), and from the *H* compartment to the *R* compartment, ($dHR_t$), we decided to use the discrete-time approximation to the stochastic continuous-time SEIQRHD model defined in Eq. (1) with the following assumptions:

1. $$dQR_t = \gamma _Q Q_{t-1}$$, $$t\ge 12$$
2. $$dHR_t = \displaystyle \frac{\omega }{1-\omega } {dQR_{t}}$$, $$t\ge 12$$
3. $$dQH_t = (H_t - H_{t-1} + dHD_t + dHR_t)I_{(H_t - H_{t-1} + dHD_t + dHR_t\ge 0)}$$, $$t\ge 13$$ ;

where $I_{(H_t - H_{t-1} + dHD_t + dHR_t\ge 0)}$ is an indicator function taking the value 1, if the argument $H_t - H_{t-1} + dHD_t + dHR_t \ge 0$ is true, and zero otherwise, $\gamma _{Q}>0$ and $0<\omega <1$.

The first assumption originates from the fact, already mentioned, that the recovery rate is roughly constant for small time intervals. The second assumption originates from the empirical evidence that about 80% of COVID-19 patients recover from the disease without needing special treatment, and for the majority—especially for children and young adults—illness due to COVID-19 is generally minor. However, for some people it can cause serious illness (20%)—difficulty in breathing requiring hospital care—which is particularly true for people who are aged over 60 years and people who have underlying medical conditions such as diabetes, heart disease, respiratory disease or hypertension. Therefore, assuming that $0<\omega <1$ of the total outgoing flows from $Q_t$ go to $H_t$ we have:

3 $$\begin{aligned} dHR_t= & {} \omega dR_t,\; 0<\omega <1 \Leftrightarrow \nonumber \\ dHR_t= & {} \omega (dHR_t+dQR_t) \Leftrightarrow \nonumber \\ dHR_t= & {} \frac{\omega }{1-\omega }dQR_t, \end{aligned}$$

The third assumption relies on the fact that, having calibrated the values of $dHR_t$ and using the observed values of $H_t$, *dQH* is calibrated by difference, provided that results in a non-negative flow.

The flows described in the assumptions are only used for $t \ge 13$, and $t \ge 12$, because the number of hospitalised individuals, $H_t$, is equal to the observed cumulative number of quarantined cases in the first 12 days of the epidemic, $h_t=\sum _{i=1}^t dIQ_i,\; t=1,...,12$ and we assume no recoveries in the first 11 days of the epidemic.

Day 84th of the epidemic corresponds to May 24th, 2020, the time point where 9844 recovered cases were released regarding recoveries that happened prior to May 24th, 2020. To calibrate the aforementioned flows the following algorithm is implemented as:

1. For the period $t=1$ to $t=84$ (May 24th 2020):
  Initialise $${\hat{S}}_1=s_1$$, $${\hat{E}}_1=e_1$$, $${\hat{I}}_1=i_1$$, $${\hat{Q}}_1=q_1$$, $${\hat{H}}_1=0$$, $${\hat{R}}_1=0$$, $${\hat{D}}_1=0$$.
  for $$t=2$$ to *T*
    1. $${\hat{S}}_t = {\hat{S}}_{t-1} - {\widehat{dSE}}_t$$
    2. $${\hat{E}}_t = {\hat{E}}_{t-1} + {\widehat{dSE}}_t - {\widehat{dEI}}_t$$
    3. $${\hat{I}}_t = {\hat{I}}_{t-1} + {\widehat{dEI}}_t - {\widehat{dIQ}}_t$$
    4. $${\widehat{dQR}}_t = round(\gamma _{0_Q}(1-\exp (-\rho _{\gamma _Q}t)){\hat{Q}}_{t-1}) I_{(t \ge 13)}+0$$
    5. $${\widehat{dHR}}_t = round(\omega \exp (-\rho _\omega t){\widehat{dQR}}_t)I_{(t \ge 12)}+0$$
    6. $${\widehat{dHD}}_t = dD_t$$
    7. $${\widehat{dQH}}^{(1)}_t =(H_t - H_{t-1} + {\widehat{dHD}}_t + {\widehat{dHR}}_t)I_{(H_t - H_{t-1} + {\widehat{dHD}}_t + {\widehat{dHR}}_t\ge 0)}I_{(t \ge 13)}+0$$
    8. $${\hat{Q}}_t = {\hat{Q}}_{t-1} + {\widehat{dIQ}}_t - {\widehat{dQH}}_t - {\widehat{dQR}}_t$$
    9. $${\hat{H}}_t = {\hat{H}}_{t-1} + {\widehat{dQH}}_t - {\widehat{dHR}}_t - {\widehat{dHD}}_t$$
    10. $${\widehat{dQH}}^{(2)}_t = ({\widehat{dQH}}_t - ({\hat{H}}_t - H_t)I_{({\hat{H}}_t- H_t>0)})I_{({\widehat{dQH}}_t - ({\hat{H}}_t-H_t)I_{({\hat{H}}_t- H_t>0)})>0)}I_{(t \ge 13)}+0$$
    11. $${\hat{R}}_t = {\hat{R}}_{t-1} + {\widehat{dQR}}_t + {\widehat{dHR}}_t$$
    12. $${\hat{D}}_t = {\hat{D}}_{t-1} + {\widehat{dHD}}_t$$
    13. $${\hat{C}}_t = {\hat{C}}_{t-1} + {\widehat{dSE}}_t$$
    14. $${\hat{A}}_t = {\hat{A}}_{t-1} + {\widehat{dIQ}}_t - {\widehat{dHD}}_t - {\widehat{dHR}}_t - {\widehat{dQR}}_t$$
2. For the remaining period ($t=85$ to $t=T$) we run the previous algorithm for every disjoint time frame of 7 consecutive days, initialising the values of each compartment with the final values of the previous period, allowing for different estimates of $\gamma _Q$ and $\omega$ across time.

The “hat” in the above notation denotes calibrated figures (either on the first or the second procedure steps) whilst the figure with no “hat” are currently observed. The calibrated value ${\widehat{dQH}}^{(1)}_t$ (7th step) is submitted to a second verification in the 10th step of the algorithm to avoid flows into compartment *H* that are higher than actually observed. Indeed, if the calibrated value ${\hat{H}}_t$ is higher than the observed value $H_t$ we reduce the inflow into $H_t$, ${\widehat{dQH}}^{(1)}_t$, by the difference ${\hat{H}}_t - H_t$, provided this reduction produces a non-negative value, ${\widehat{dQH}}^{(2)}_t$.

In evolutionary computation, differential evolution (DE)^36^ is a method that optimises a problem by iteratively trying to improve a candidate solution with regard to a given measure of quality. Such methods are commonly known as meta-heuristics as they make few or no assumptions about the problem being optimised and can search very large spaces of candidate solutions. DE is used for multidimensional real-valued functions but does not use the gradient of the problem being optimised, which means DE does not require the optimisation problem to be differentiable, as is required by classic optimisation methods such as gradient descent and quasi-Newton methods. Therefore, DE can also be used on optimisation problems that are not even continuous, are noisy, change over time, etc.. DE optimises a problem by maintaining a population of candidate solutions and creating new candidate solutions by combining existing ones according to its simple formulae, and then keeping whichever candidate solution has the best score on the optimisation problem at hand. Using a DE algorithm (provided by “R” package “DEoptimR”), for the first period ($t=1$ to $t=84$) we minimise the loss function:

4 $$\begin{aligned} {{\mathcal {L}}}_1(\varvec{\theta })= \sum _{t=1}^{t^f}(H_{t}-{\hat{H}}_{t})^2 + (R_{84}-{\hat{R}}_{84})^2 + \nonumber (R^{(Q)}_{84}-9652)^2. \end{aligned}$$

and for the remaining sets of 7-day periods we minimise the loss function:

5 $$\begin{aligned} {{\mathcal {L}}}_2(\varvec{\theta }) = \sum _{t=1}^{t^f}\left( (H_{t}-{\hat{H}}_{t})^2+(R_{t}-{\hat{R}}_{t})^2\right) \end{aligned}$$

The first row of loss function (4) ensures the calibrated daily hospital occupancy is equal to the observed daily hospital occupancy. The second row ensures that the calibrated number of recovered individuals on the correction (day 84th) matches the observed and reliable figure as closely as possible. The third row ensures the accumulated number of recovered individuals originated on the quarantined compartment, $R^{(Q)}_t=\sum _{i=1}^t dQR_i$, is equal to the number of recovered individuals recorded by the Portuguese primary health care system, but released at once on May 24th, 2020 (9652 individuals). The second loss function (5) ensures the calibrated daily hospital occupancy is equal to the observed daily hospital occupancy and the daily accumulated number of recovered individuals is equal to the daily accumulated number of recovered individuals.

This procedure allows the calibrations of the flows $dQH_t$, $dQR_t$ and $dHR_t$, conditional to the flows $dSE_t$ and $dEI_t$ calibrated in the first place, as described above.

The upper and lower limits of the hypersquare where the best solution is searched for are:

- $$(\gamma _{Q}^{(u)},\eta ^{(u)},h_0^{(u)},h_1^{(u)},\rho _h^{(u)}, \tau ^{(u)})=(1/10,0.999)$$ and
- $$(\gamma _{Q}^{(l)},\eta ^{(l)},h_0^{(l)},h_1^{(l)},\rho _h^{(l)}, \tau ^{(l)})=(1/120,0.001)$$.

The initial conditions are the two calibrated values in step one, $e_1=dSE_1$ and $i_1=dEI_1$ and the observed value $q_1=dIQ_1$ and $s_1=N-e_1-i_1-q_1$.

**Figure 7:**
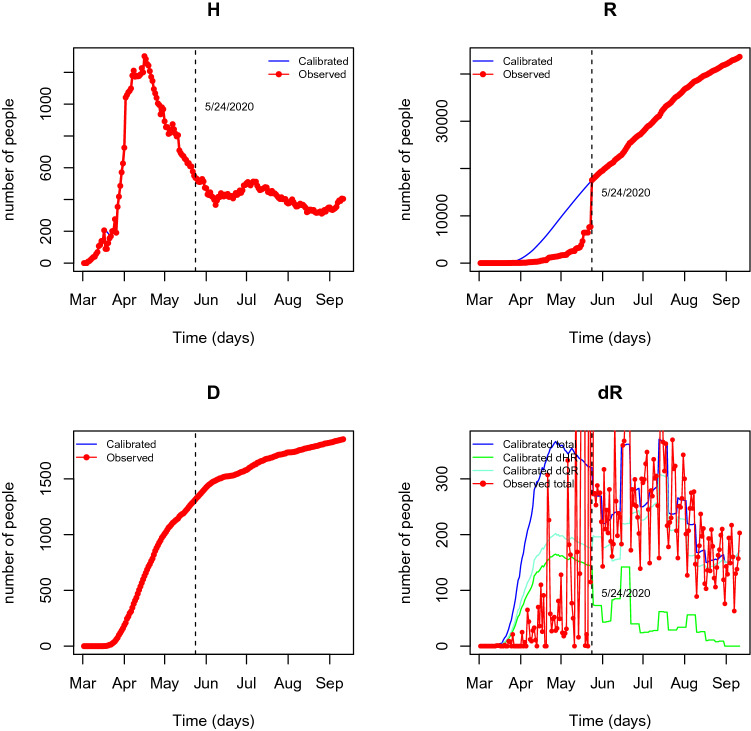
Observed and calibrate course of compartments H, R and D, and flows coming into compartment R.

The calibrated values *H*, *R*, and *D* compartments resulting from the second step of the calibration procedure along with the original observed data are represented in panels (H), (R) and (D) of Fig. 7. The calibrated flows ${\widehat{dQR}}_t$, ${\widehat{dHR}}_t$ and ${\widehat{dR}}={\widehat{dQR}}_t+{\widehat{dHR}}_t$ are represented in Fig. 7 (dR) along with the observed total flow *dR* (the deviation between the observed and calibrated *dR* on 2020-09-27, the last day under analysis, is red 0.3%). As expected, the accumulated number recovered individuals on May 25th (day 85) is interpolated, back-casting the excess of recovered cases released on May 24th (day 84), accordingly to the interdependence model dynamics. As such, the curve of calibrated quarantined individuals reproduces almost exactly its course after May 24th and flattens before this day, reproducing the calibrated recovered flows ($dQR_t$). This last flow, jointly with the flow of recovered individuals from the hospitalised compartment, $dHR_t$ are calibrated resulting in reliable courses both for accumulated recovered individuals and daily recovered individuals.

### Bayesian hierarchical modelling

Using the discrete-time approximation to the stochastic continuous-time SEIQRHD model (2) defined above we set up a Bayesian hierarchical model where the incidence variables are assumed stochastic. The transitions of individuals from the one compartment to the next one of the SEIQRHD model are considered stochastic movements between the corresponding population compartments. In each period an individual either stays in or moves on to the next compartment. In reliability analysis life-time is usually considered to follow an exponential distribution. By analogy, here the time length that an individual spends in a compartment is exponentially distributed with some compartment-specific rate $\lambda _t$. Therefore, the probability of extending the stay by a further period of length *h* is $\exp (-\lambda _t h)$ and the probability of leaving is therefore $1 - \exp (-\lambda _t h)$. The summation over the individual Bernoulli trials assuming they are independent and identical for all members of a compartment would result in binomial distributions^37^. Due to the scale we are working with, we found it useful to take advantage of the approximation of the binomial to the Poisson distribution. Therefore incidences are assumed to follow the distributions:

6 $$\begin{aligned} \begin{array}{lll} dSE_t &{} \sim &{} Pois(S_t \times p_{dSE}(t)),\\ dEI_t &{} \sim &{} Pois(E_t \times p_{dEI}),\\ dIQ_t &{} \sim &{} Pois(I_t \times p_{dIQ}),\\ dQH_t &{} \sim &{} Pois(Q_t \times h_t \times p_{dQH}),\\ dQR_t &{} \sim &{} Pois(Q_t\times (1-h_t)\times p_{dQR}(t)),\\ dHR_t &{} \sim &{} Pois(H_t\times (1-\mu )\times p_{dHR}),\\ dHD_t &{} \sim &{} Pois(H_t\times \mu \times p_{dHD}), \end{array} \end{aligned}$$

where the transition probabilities are given by:

7 $$\begin{aligned} \begin{array}{lll} p_{dSE}(t) &{} = &{} 1- \exp \left( -\beta \frac{I_t}{N}\right) ,\\ p_{dEI} &{} = &{} 1- \exp \left( -\delta \right) ,\\ p_{dIQ} &{} = &{} 1- \exp \left( -\kappa \right) ,\\ p_{dQH} &{} = &{} 1- \exp \left( -\eta \right) ,\\ p_{dQR}(t) &{} = &{} 1- \exp \left( -\gamma _Q(t)\right) ,\\ p_{dHR} &{} = &{} 1- \exp \left( -\gamma _H\right) ,\\ p_{dHD} &{} = &{} 1- \exp \left( -\alpha \right) \end{array} \end{aligned}$$

assuming the time unit *h* is 1 day.

Conditional on all information up to time *t*, the Poisson random variables $dSE_t$, $dEI_t$, $dIQ_t$, $dQH_t$, $dQR_t$, $dHR_t$ and $dHD_t$ are independent. The model further assumes that the population size *N* remains constant and that individuals mix homogeneously. In order to account for the effects of control measures such as lockdown and social distancing, we assume that the transmission parameter $\beta$ is constant up to the time point when the control measures are introduced and after that decays exponentially at a constant rate. During the SARS-CoV-2 epidemic in Portugal four distinct periods can be distinguished. The first one, between the beginning of the epidemic and the declaration of the *state of emergency*, the first hard lockdown control measures (on March 18th 2020) where the transmission parameter is assumed to be constant. The second one, between March 18th, 2020, and the declaration of the *situation of calamity* when the lockdown measures started easing (May 4th, 2020). A third period from May 4th onwards and until August 18th, corresponding to the traditional Portuguese holidays time and the beginning of the school year and finally, from August 18th onwards. This is formulated as:

8 $$\begin{aligned} \beta (t)=\left\{ \begin{array}{ll} \beta _0, &{} t<\tau _0\\ \beta _0 e^{-\rho _{\beta _0}(t-\tau _0)}, &{} \tau _0 \le t< \tau _1,\\ \beta _1 e^{-\rho _{\beta _1}(t-\tau _1)}, &{} \tau _1 \le t < \tau _2,\\ \beta _2 e^{-\rho _{\beta _2}(t-\tau _2)}, &{} t\ge \tau _2 \end{array}\right. \end{aligned}$$

where $\tau _0$, $\tau _1$ and $\tau _2$ correspond to those three intervention dates (March 18th, May 4th, and August), $\beta _0$ is the initial transmission rate, and $\rho _{\beta _0}, \rho _{\beta _1}, \rho _{\beta _2} > 0$ are the rates at which $\beta _0$, $\beta _1$ and $\beta _2$ decay on $\tau _0 \le t < \tau _1$, $\tau _1 \le t < \tau _2$ and $t \ge \tau _2$, respectively.

The basic reproduction number $R_0$ is defined as the average number of secondary cases generated by a primary case over his/her infectious period when introduced into a large population of susceptible individuals^38^. The constant $R_0$ thus measures the initial growth rate of the epidemic and for the model above is $R_0 = \displaystyle \frac{\beta _0}{\kappa }$^39^. Furthermore^40^, define the time dependent effective reproductive number $R_0(t) = \displaystyle \frac{\beta _t}{\kappa }\frac{S_t}{N}$ as the number of secondary cases per infectious case at time *t*. Because $S_t \approx N$, it follows that $R_0(t) = \displaystyle \frac{\beta _t}{\kappa }$ is a function proportional to the time-varying transmission rate in (8). The time point at which $R_0(t)$ assumes values smaller than 1 indicates when control measures have become effective in controlling the epidemic.

The parameter $\kappa$ governs the passage from the infective to the quarantined compartment, as explained before. An infected individual is not automatically quarantined, because the authorities are often unable to test enough people while keeping pace with the spread. This measure is especially difficult because many people do not develop symptoms at all, but can transmit the infection to others. So, we believe that $\kappa$ also contains some information about the percentage of the detected infective people. A study by^40^ proposed the introduction of an additional parameter to understand this issue better. Despite all these considerations, we used values estimated from a microdata file provided by DGS in the first step of data calibration (as explained before). Therefore, we decided to move on a parsimonious parameterisation for $\kappa$, considering it fixed and equal to 1/4.73 days (see Table 1). Similarly, the incubation period, $\delta ^{-1}$ is considered fixed and equal to 6.5 days (see Table 1). Accordingly to what we have discussed before, we assume $\eta$ is constant, and $h_t$ follows the model:

9 $$\begin{aligned} h(t)= h_{0}\exp (-\rho _h t). \end{aligned}$$

The parameters $\gamma _Q$ and $\gamma _H$ govern the passage from the *Q* and *H* compartments to the *R* compartment. As already mentioned, we assume $\gamma _Q$ is much more dependent on protocols for recovery confirmation and therefore much more susceptible to increase over time than $\gamma _H$. Therefore, $\gamma _Q$ follows an exponential trend, whilst $\gamma _H$ is considered fixed. The assumption is that the recovery rate, $\gamma _Q(t)$ converges towards an asymptotic value:

10 $$\begin{aligned} \gamma _Q(t)= \gamma _{0_Q}(1-\exp (-\rho _\gamma t)). \end{aligned}$$

The value of $\gamma _{0_Q}$ is the final asymptotic value of the recovery rate. It depends on $\rho _\gamma$ which represents how fast the health system learns to respond to the disease and the protocol on recovery confirmation evolves.

Finally, the parameter $\mu$, which governs the passage from the *H* compartment to the *D* compartment, corresponds to the share of the hospitalised people who dies due to the disease. Again, we believe $\mu$ is related to how a health system can improve its capability to treat people over time. Nevertheless, for parsimonious reasons and given the residual hospitalised fatality rate of COVID-19 we decided to assume $\mu$ is fixed. Evoking the aforementioned reasons, the parameter $\alpha$ is also considered fixed, but highly variable between individuals.

The epidemic model specified in (2), (6) and (7), together with the transmission rate model (8) and models (9) and (10) and the parameters $\delta$, $\kappa$, $\eta$, $\gamma _H$, $\mu$ and $\alpha$ has parameter vector $\varvec{\theta }= (\beta _0, \beta _1, \rho _{\beta _0},\rho _{\beta _1}, \delta ,\kappa , \eta , h_0, h_1, \rho _h, \tau , \gamma _{0_Q}, \rho _\gamma , \gamma _H,\mu , \alpha )$, which we would like to estimate from the knowledge of initial conditions $S_0 = s_0$, $E_0 = e_0$, $I_0 = i_0$, $Q_t=q_0$ $R_0 = 0$, $H_t=0$ and $D_0=0$, population size, *N*, and from observed values of ${dSE_t, dEI_t, dIQ_t, dQH_t, dQR_t, dHR_t, dHD_t}$.

Gamma and uniform priors are assigned to each of the parameters in $\varvec{\theta }$ as

11 $$\begin{aligned} \begin{array}{llllll} \beta _0 &{} \sim &{} \text {Ga}(2,10), &{} \beta _1 &{} \sim &{} \text {Ga}(2,10),\\ \beta _2 &{} \sim &{} \text {Ga}(2,10), &{} \rho _0 &{} \sim &{} \text {Ga}(10,100),\\ \rho _1 &{} \sim &{} \text {Ga}(10,100), &{} \rho _2 &{} \sim &{} \text {Ga}(10,100),\\ h_0 &{} \sim &{} \text {Unif}(0,1), &{} \rho _h &{} \sim &{} \text {Ga}(1,10),\\ \gamma _{0_Q} &{} \sim &{} \text {Ga}(10,100), &{} \rho _\gamma &{} \sim &{} \text {Ga}(1,10),\\ \eta &{} \sim &{} \text {Ga}(2,10), &{} \gamma _H &{} \sim &{} \text {Ga}(10,100),\\ \mu &{} \sim &{} \text {Unif}(0,1), &{} \alpha &{} \sim &{} \text {Ga}(10,100) \end{array} \end{aligned}$$

where $\text {Ga}(a,b)$ refers to a gamma distribution with parameters shape *a* and rate *b*, mean *a*/*b*, and variance $a/b^2$ and $\text {Unif}(a,b)$ to a uniform distribution between *a* and *b* with a mean $(a+b)/2$ and variance $1/(12(b-a))$.

Let $\mathbf {dSE}=\{dSE_t, t=1,2,...,T\}$ be the daily observed (calibrated) counts of susceptible individuals who become infected. We define similarly the other vectors: $\mathbf {dEI}$, $\mathbf { dIQ}$, $\mathbf {dQH}$, $\mathbf {dQR}$, $\mathbf {dHR}$ and $\mathbf {dHD}$. Because the series are conditionally independent the likelihood of the data is:

12 $$\begin{aligned} {{\mathcal {L}}}= & {} p(\mathbf {dSE}| \cdot )p(\mathbf {dEI}| \cdot )p(\mathbf {dIQ}| \cdot )p(\mathbf {dQH}| \cdot )p(\mathbf {dQR}| \cdot )p(\mathbf {dHR}| \cdot )p(\mathbf {dHD}| \cdot ) \nonumber \\= & {} \prod _{t=1}^T p(dSE_t| \cdot )p(dEI_t| \cdot )p(dIQ_t| \cdot )p(dQH_t| \cdot ) \times \nonumber \\&p(dQR_t| \cdot )p(dHR_t| \cdot )p(dHD_t| \cdot ), \end{aligned}$$

where $p( \cdot | \cdot )$ stands for the Poisson transition probabilities specified in (6) conditioned on $\varvec{\theta }$ and on all the information up to time *t*. Given the hierarchical representation presented above and using the same notation, one can evaluate the posterior distribution of all of the parameters, given the observed counts:

13 $$\begin{aligned} \Pi (\varvec{\theta }|\mathbf {dSE},\mathbf {dEI},\mathbf {dIQ},\mathbf {dQH},\mathbf {dQR},\mathbf {dHR},\mathbf {dHD}). \end{aligned}$$

One cannot evaluate this posterior distribution analytically and must resort to numeric simulation methods. We use the special case of MCMC known as Gibbs sampling^41^ and implement the algortithm using the “R” package “JAGS” (all code used in this paper can be obtained from authors upon request).

## Results

After a burn-in period of 10,000 iterations by which we believe convergence has been achieved, a sample of size 250 taken every 500 iterations (to avoid serial correlation, especially for parameters where identification problem may arise, such as $\eta$ and *h* and $\mu$ and $\alpha$) of the chain was used to obtain marginal posterior distributions for all model parameters.

Convergence of the Markov chain was assessed using a series of runs for four different chains with different starting values and also inspecting the autocorrelation function (cf. Figures 15, 16, 17 and 18 in Supplementary Material). In all cases the Markov chain appeared to have converged after the burn-in period.

**Figure 8:**
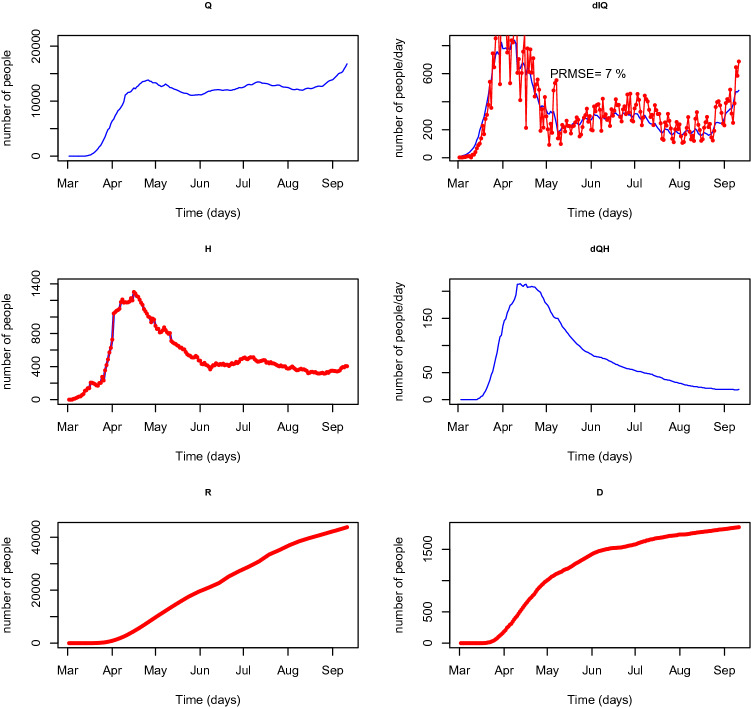
Results of SEIQRHD model. Means of the posterior predictive distributions of the most important flows for pandemic course management are represented in blue lines. Red lines represent compartments or flows for which observed values exist. The percentual root mean square error (PRMSE) is indicated in panel *dIQ* for the central flow that drives the course of the epidemic.

Analysing the fit of the model against real data is relevant as a tool of external validation. Therefore, the model fit is assessed by plotting the available data against the estimated expected values of the compartments. The fit of the model is evident from Fig. 8 for the period corresponding to the used data. Among the model variables represented, the flow of positive cases that are isolated, *dIQt*, is of essential importance, as is the one that drives the course of the epidemic and captures most of the attention of the health authorities. Thus, we have assessed the predictive relevance of the model by calculating the root mean square error of prediction (represented in the percentage of the mean of *dIQt*). The resulting value of 7% allows to confirm the validity of the model. Moreover, it is possible to assess the daily number of people in need of medical care through the flow *dQH* which is not released by the health authorities.

Table 2 presents the main results for the model parameters.

**Table 2:** Posterior statistics of SEIQRHD model parameters.

Parameter	Description	Units	Prior mean (std)	Posterior mean (std)	Days (95% CrI)
$R_0$	Basic infection reproduction number	No.	–	3.424 (0.0610)	–
$\beta _0$	Contact rate before first intervention	days$^{-1}$	0.2 (0.1414)	0.724 (0.0130)	–
$\beta _1$	Contact rate on second intervention	days$^{-1}$	0.2 (0.1414)	0.252 (0.0051)	–
$\beta _2$	Contact rate on third intervention	days$^{-1}$	0.2 (0.1414)	0.402 (0.0174)	–
$\rho _{\beta _0}$	Rate of decay of $\beta _0$	days	0.1 (0.0316)	0.007 (0.0012)	–
$\rho _{\beta _1}$	Rate of decay of $\beta _1$	days	0.1 (0.0316)	0.006 (0.0004)	–
$\rho _{\beta _2}$	Rate of decay of $\beta _2$	days	0.1 (0.0316)	0.016 (0.0038)	–
$\eta$	Hospitalisation rate	days$^{-1}$	0.2 (0.1414)	0.636 (0.1349)	1.13–2.69
$h_0$	Initial probability of hospitalisation	%	0.5 (0.2887)	0.067 (0.0116)	–
$\rho _h$	Rate at which probability of hospitalisation decreases	%	0.1(0.1)	0.018 (0.0002)	–
$\gamma _0$	Final (asymptotic) recovery rate of quarantined people	days$^{-1}$	0.01 (0.0032)	0.016 (0.0001)	62.89–64.43
$\rho _\gamma$	Rate at which recovery rate of quarantined people incresases	days	0.1 (0.0316)	0.081 (0.0106)	–
$\gamma _H$	Recovery rate of hospitalised individuals	days$^{-1}$	0.01 (0.0032)	0.180 (0.0051)	5.24–5.85
$\mu$	Hospitalised case fatality rate	%	0.5 (0.2887)	0.26 (0.0177)	–
$\alpha$	Rate of death	days$^{-1}$	0.01 (0.0032)	0.07 (0.0052)	12.12–16.31

**Figure 9:**
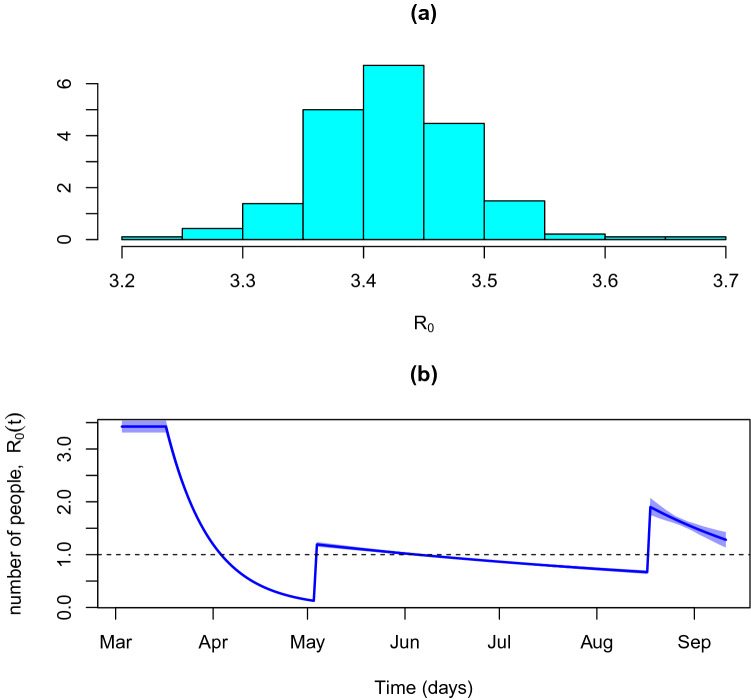
Infection reproduction number. (**a**) Basic Reproduction Number distribution, $R_0$. (**b**) Effective reproduction number, $R_0(t)$.

The mean of the basic reproduction number ($R_0$) is 3.434 with a 95% credible interval of (3.312–3.535) and varies between 3.250 and 4.165. The distribution of the basic reproduction number is depicted in Fig. 9a. The temporal evolution of the effective reproduction number is represented in Fig. 9b. The imposition of the lockdown and social distancing measures were effective in pulling the reproduction number down to a value below the epidemic control of one. Nevertheless, easing those measures implied a significant increase on the infection transmission in early May. Still, the level that was reached kept the disease’s spread under control and additional restrictions made it possible to bring it to levels close to the threshold, although the end of the epidemic cannot be anticipated yet.

**Figure 10:**
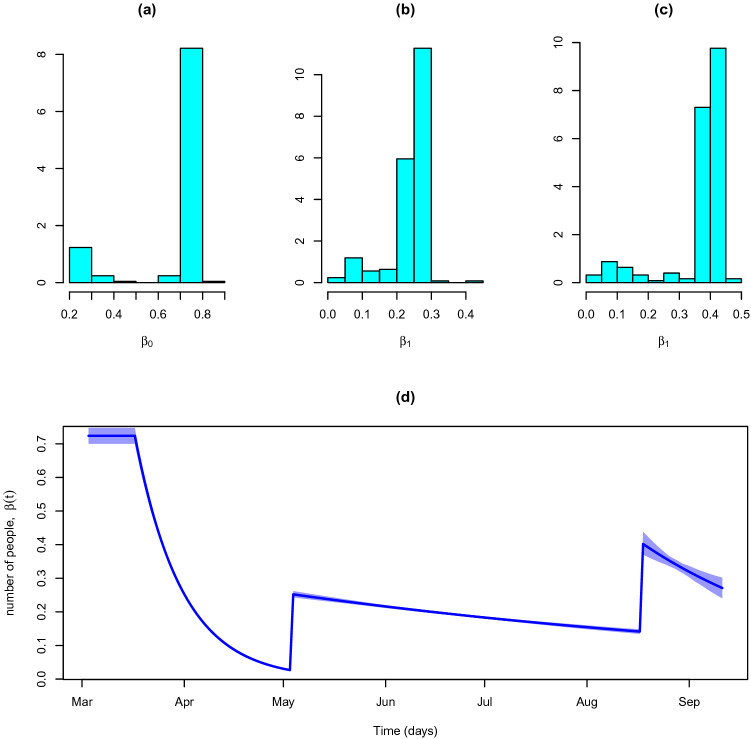
Transmission rate, $\beta _t$. (**a**) Initial transmission rate distribution, $\beta _0$. (**b**) Transmission rate distribution after the second intervention, $\beta _1$. (**c**) Transmission rate distribution after the third intervention, $\beta _2$. (**d**) Course of the transmission rate, $\beta (t)$.

The posterior mean of transmission rate before first intervention ($\beta _0$) is 0.724 with a 95% credible interval of (0.700–0.747). These values are consistent with the values published in the literature (see, for example,^20^). By the time the second intervention takes place, the value of $\beta (t)$ is only 0.027 (0.025–0.029). This explains the benefits of imposing lockdown measures to bring the transmission rate down from 0.724 to 0.027. As already mentioned, on May 4th the lockdown measures started to be eased and the transmission rate raised to 0.252 (0.243–0.262) starting a new downward path. Nevertheless, the end of the Portuguese traditional holidays period and (second half of August) and the beginning of the academic year push the transmission rate up again from 0.141 to 0.402 due to the natural increase of daily contacts. The distribution of $\rho _{\beta _0}$, $\rho _{\beta _1}$, and $\rho _{\beta _2}$ are Gaussian-like with posterior means 0.07 (0.068–0.072), 0.006 (0.005–0.006), and 0.016 (0.009–0.024). The rate of decay of $\beta _1$ is very minimal. Indeed, the value of $\beta (\tau _2-1)$ (the day before of the third intervention) is 0.14 indicating the transmission rate after the second intervention (0.24) has a small decay. Actually, although the current mobility levels (cf. *Google Mobility Reports*) are below the pre-pandemic recorded figures, from early May onwards they started to catch up and are currently close to the pre-pandemic levels from early May onwards, potentially inducing a large number of daily infections. The posterior distribution of the transmission rates, $\beta _0$, $\beta _1$ and $\beta _2$ are represented in Fig. 10a–c. The temporal evolution of the transmission rate is represented in Fig. 10d.

**Figure 11:**
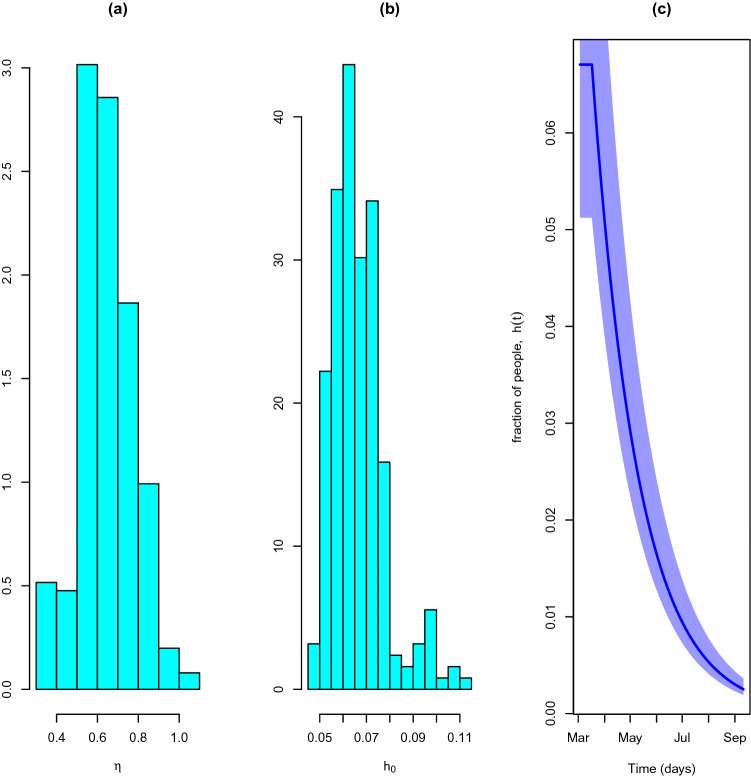
Hospitalisation rate. (**a**) Hospitalisation rate distribution, $\eta$. (**b**) Initial hospitalisation fraction, $h_0$. (**c**) Hospitalisation fraction course, *h*(*t*).

The posterior mean of the hospitalisation rate ($\eta$) is between 0.636 (0.371–0.888) corresponding to a mean period of 1.13–2.69 days between case confirmation and hospital admittance. The posterior mean of the initial hospitalisation probability $h_0$ is 6.71% (5.12–9.89%). Confirming what is currently known about the severity of the disease this figure indicates that only a small proportion of the quarantined individuals need specialised medical care. The posterior distributions of the hospitalisation rate, $\eta$, and initial hospitalisation fraction, $h_0$ are represented in Fig. 11a,b. The temporal evolution of the hospitalisation fraction is represented in Fig. 11c.

**Figure 12:**
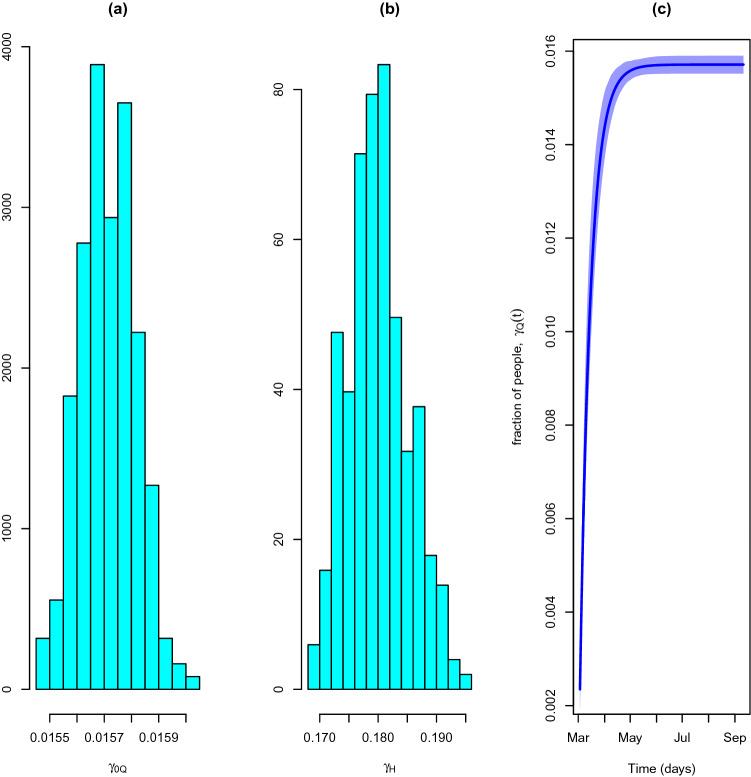
Recovery rate. (**a**) Asymptotic recovery rate for quarantined individuals’ distribution, $\gamma _{0_Q}$. (**b**) Recovery rate for hospitalised individuals’ distribution, $\gamma _H$. (**c**) Recovery rate course for quarantined individuals, $\gamma _{Q}(t)$.

Regarding recovery, we observe a mean asymptotic recovery rate of 0.016 (0.0155–0.0159). This rate translates in a mean asymptotic recovery period of 63.6 (62.89–64.43) days. Although it seems to be larger than expected, this value is affected by the speed of the reporting of recovered individuals. Indeed, it is consensual between epidemiologists in Portugal that recovered individuals figures released by DGS have been permanently below the expert expectations, given recovery time reported in the literature (around 14 days, on average) and admitted by clinical practitioners. Indeed, by the time we of finishing this manuscript DGS has already released a batch of additional 13529 recovered individuals (referring to the previous period with unspecified allocation) that would result in a reduction of the recovery time consistent with the usual clinical mean recovery period. The posterior distribution of the asymptotic recovery rate is depicted in Fig. 12a. The temporal evolution of the recovery rate is represented in Fig. 12c.

On the other hand, the posterior mean of the recovery rate of hospitalised individuals is 0.18 (0.171–0.191) which corresponds to a period of 5.5 (5.24–5.85) days in hospital before recovery confirmation. That is a perfectly reasonable period despite its dependence on the health system promptness. The posterior distribution of the mean recovery rate of hospitalised individuals is depicted in Fig. 12b.

**Figure 13:**
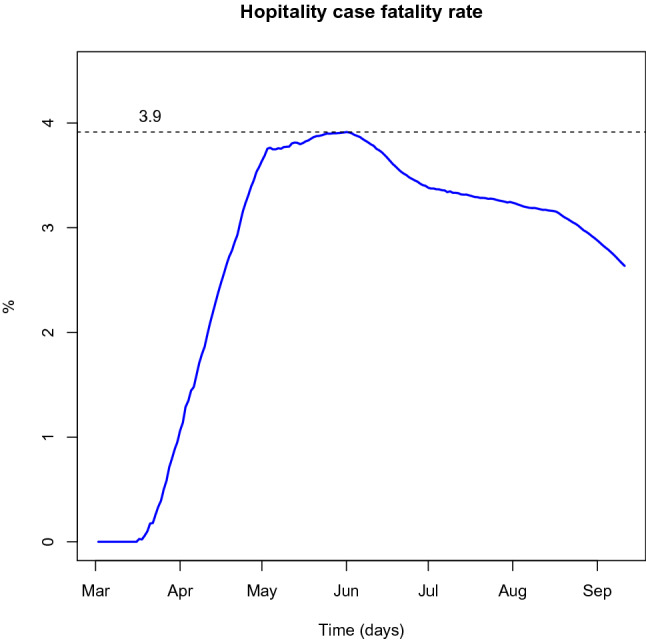
Case fatality rate.

Finally, the posterior mean of the hospitalised case fatality rate (HCFR) ($\mu$) is 26.12% (22.40–29.53%). We must remark that this HCFR concerns only the hospitalised individuals and should not be compared with the case fatality rate (CFR) only concerns the recorded number of infections. Indeed, in our model the case fatality rate is given by the ratio of $D_t$ (accumulated number of deaths) to $C_t$ (accumulated number of infected individuals). The course of the CFR is represented in Fig. 13. It follows a convergent path to a maximum value around 3.9% which is in line with the values reported by the literature, for countries that have adopted a lockdown policy for a significant period^42,43^. The posterior mean of the death rate, $\alpha$, is 0.071 (0.061–0.082) which corresponds to a period of 14.2 (12.12–16.31) days in hospital before death. As anticipated this is highly dispersed value denoting a significant heterogeneity of the length of stay in hospital among individuals.

## Discussion

This work adopts a generalised SEIR model, the so-called SEIQRHD model, to offer a quantitative overview of the complex structural analysis and prediction of the SARS-CoV-2 epidemic. The work was developed during the first wave of course of the epidemic. By the time we completed the manuscript, a new wave of increased infections was striking all over Europe.

The proposed SEIQRHD model is, to the best of our knowledge, is the first considering a specific compartment to follows the hospitalised individuals who might put an intolerable burden on the national health system, especially now that a second wave is starting and no one can anticipates the course of the epidemic when the Winter arrives. This model offers to health authorities the possibility to predict of the number of individuals who are going to require hospitalisation, thus allowing for better planning of resources. It must be emphasized that the data available to calibrate the model and discover some of the necessary flows includes infirmary and ICU occupancy, that is, the daily number of occupied beds. The calibration procedure allows to estimate the in and outflows of compartment H, thus allowing to approximate these unobserved values, whose knowledge is of extreme importance for the health system to allocate the necessary resources. Moreover, the set of model parameters (some of them ordinary in SEIR model specification), either time-fixed or time-varying, provide useful insights on the health authorities reaction to the daily challenges. Namely, it is possible to monitor (a) the time between case symptom onset and subsequently isolation, (b) the time spent in isolation, (c) the hospitalisation rate and the probability of hospitalisation and its evolution over time which provides information on the near future burden to the health system among other important insights as discussed in the section devoted to the analysis of results. It is also possible to distinguish between recovery rates and duration for hospitalised and non-hospitalised individuals, allowing a much deeper understanding on the recovery evolution and the efficiency of hospital care. Daily estimation of the SEIQRHD along with the predictions for stocks and flows between compartments may have strong implications on public health policy and resources allocation to control the pandemic. Results from the model may help health authorities to adapt their strategies and plan their resources within at least three action vectors: (a) monitor how fast the public health teams in the field identify and isolate positive cases turning possible to anticipate the number of professionals that need to be allocated to these tasks; (b) by following and predicting the hospitalization needs, allowing to anticipate possible stress levels in the healthcare provided and the reallocation of beds devoted to SARS-CoV-2 and other pathologies; (c) by better predicting the course of the pandemic in terms of incidence and prevalence, allowing a better planning of mitigation measures, including more informed decisions about the timing and intensity of confinement measures.

An additional future step over of this line of modelling is the separation between hospitalised noncritical patients from the ones in intensive care, which may of course offer authorities with additional insight for planning purposes.

Other researchers might use the methodology developed here to either calibrate non-error free or missing epidemic data. Indeed, as^44^ points out, frequently the official databases are not exempt from measurement errors and/or missing data that must be accounted for and fixed before any further analysis. Estimating missing flows or the calibrated version of observed series can be done using the rationale behind the two-step calibration procedure proposed in this paper. For Portuguese data the calibration procedure has shown to be crucial to cope with errors on the official recovery cases, with impact over virtually all the transition rates between compartments and particularly the ones related with hospital occupancy: hospitalisation rate and recovery rate from hospitalised patients.

Both the calibration methodology and the Bayesian model were adapted to the Portuguese case and corresponding available data. However, we believe the data released in Portugal, with some minor differences, are widely available in most of the developed countries. Therefore, the methodologies developed here might be successfully replicated in many other countries.

The numbers retrieved from the Portuguese data show that Portugal has been dealing with the epidemic promptly and the measures taken to flatten the incidence curve have proved to produce the desirable results. In particular, the performed modeling allowed to understand how the contact rate has evolved over time and more importantly, how effective several interventions were (lockdown or other mobility restriction measures). For example, the first Portuguese intervention allowed the transmission rate to reduce from 0.724 to 0.027, but the second intervention was much less effective only allowing a reduction from 0.24 to 0.14. It is important to notice that the first intervention corresponded to a full lockdown, while the second one corresponded to setting limited mobility restrictions. It also allows us to understand the price to pay when restrictions are removed or eased. In fact, we have observed a rapid increase of the transmission rate from 0.027 to 0.252 right before the second intervention and from 0.141 to 0.402 before the third one.

These results may help public authorities to set the timing and intensity of the measures in order to achieve the planned results and particularly to avoid the implosion of the health system.

## Supplementary Information

Supplementary Information.
