# Supplementary material for: Addressing hospitalisations with non-error-free data by generalised SEIR modelling of COVID-19 pandemic
Source: Sci Rep. 2021 Oct 4;11:19617. doi: 10.1038/s41598-021-98975-w (PMC8490474; doi:10.1038/s41598-021-98975-w)
Supplement: Supplementary file 1 — Supplementary Information. [file 41598_2021_98975_MOESM1_ESM.pdf]

Addressing hospitalisations with non-error-free data by generalised SEIR modelling of COVID-19 pandemic

immediate

A MCMC convergence diagnostics

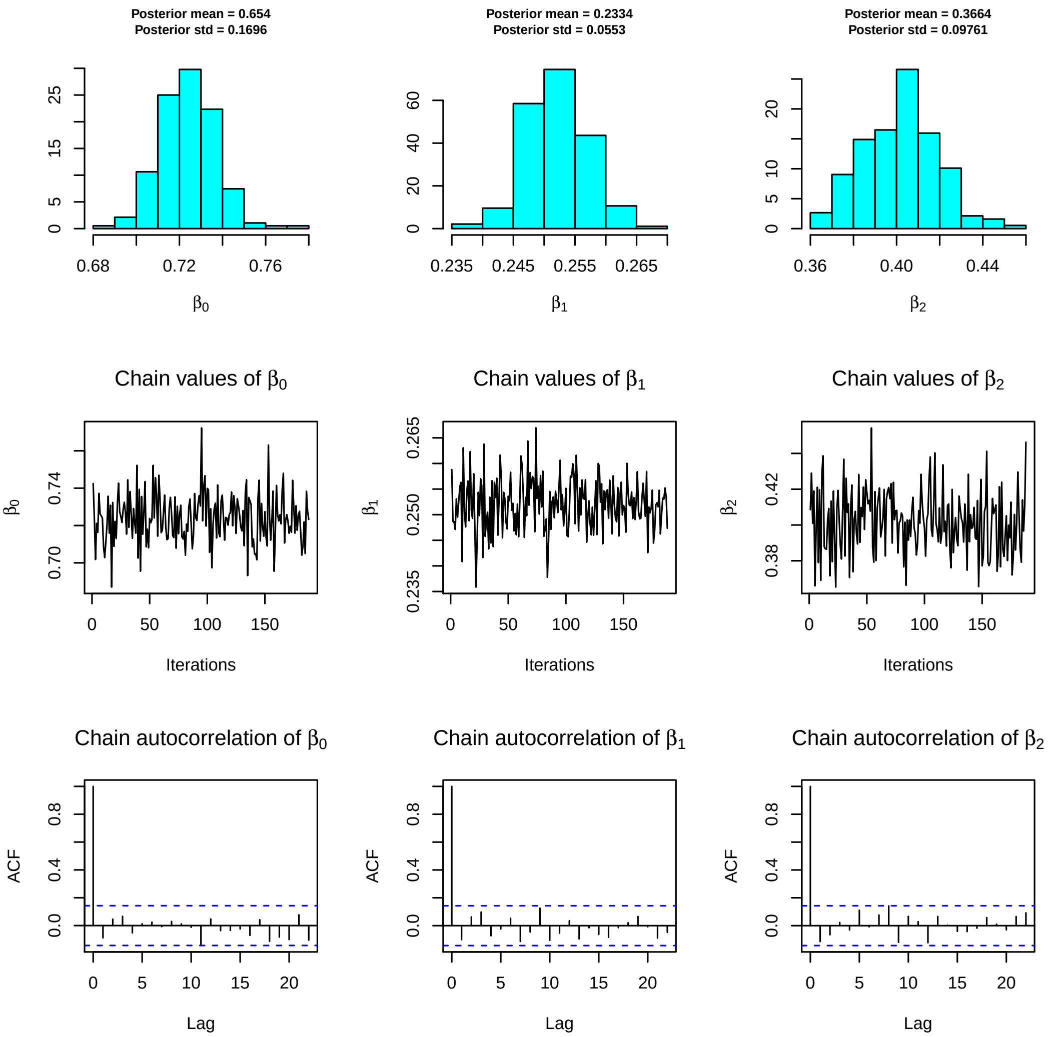

Figure 14: Posterior distribution, traceplots and autocorrelation plots of SEIQRHD model parameters β_0, β_1 and β_2.

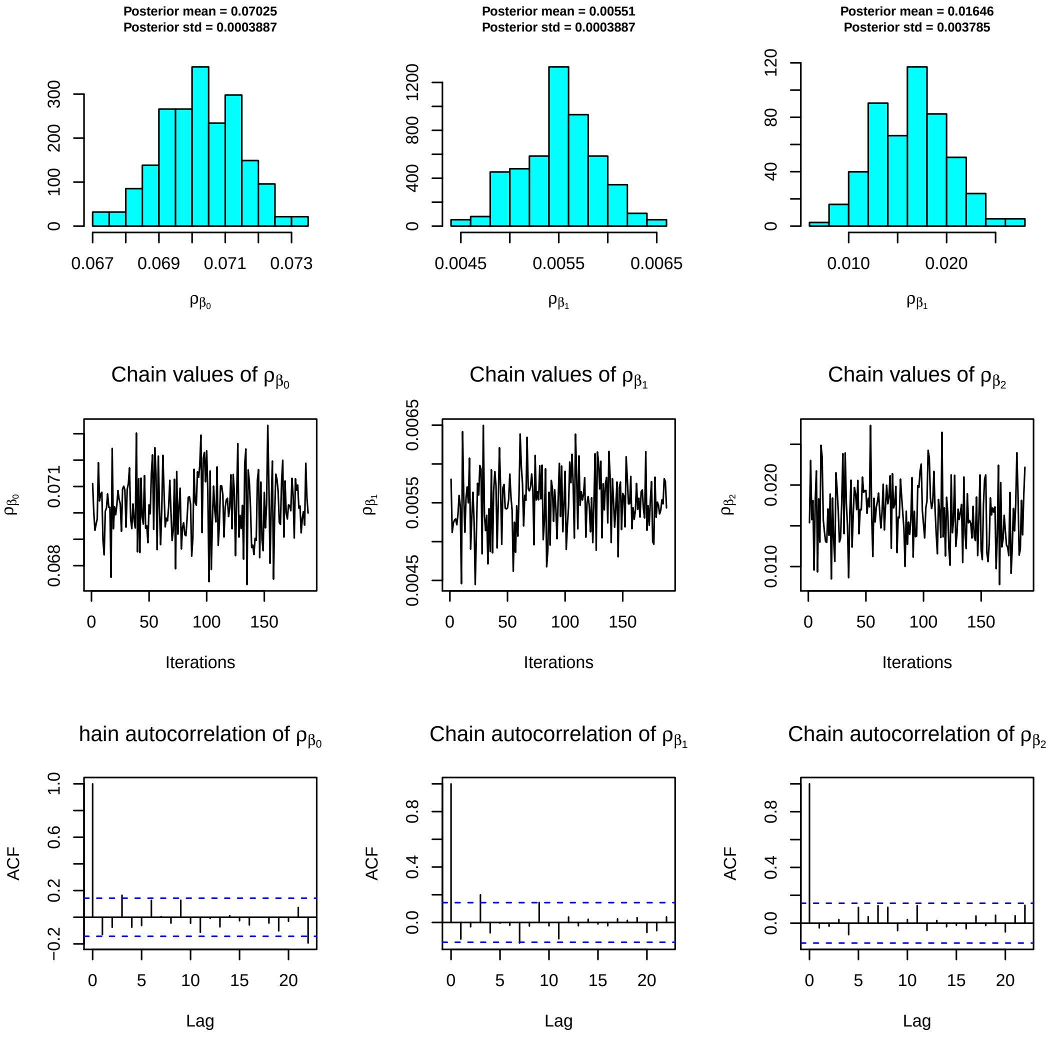

Figure 15: Posterior distribution, traceplots and autocorrelation plots of SEIQRHD model parameters ρ_{β_0}, ρ_{β_1} and ρ_{β_2}

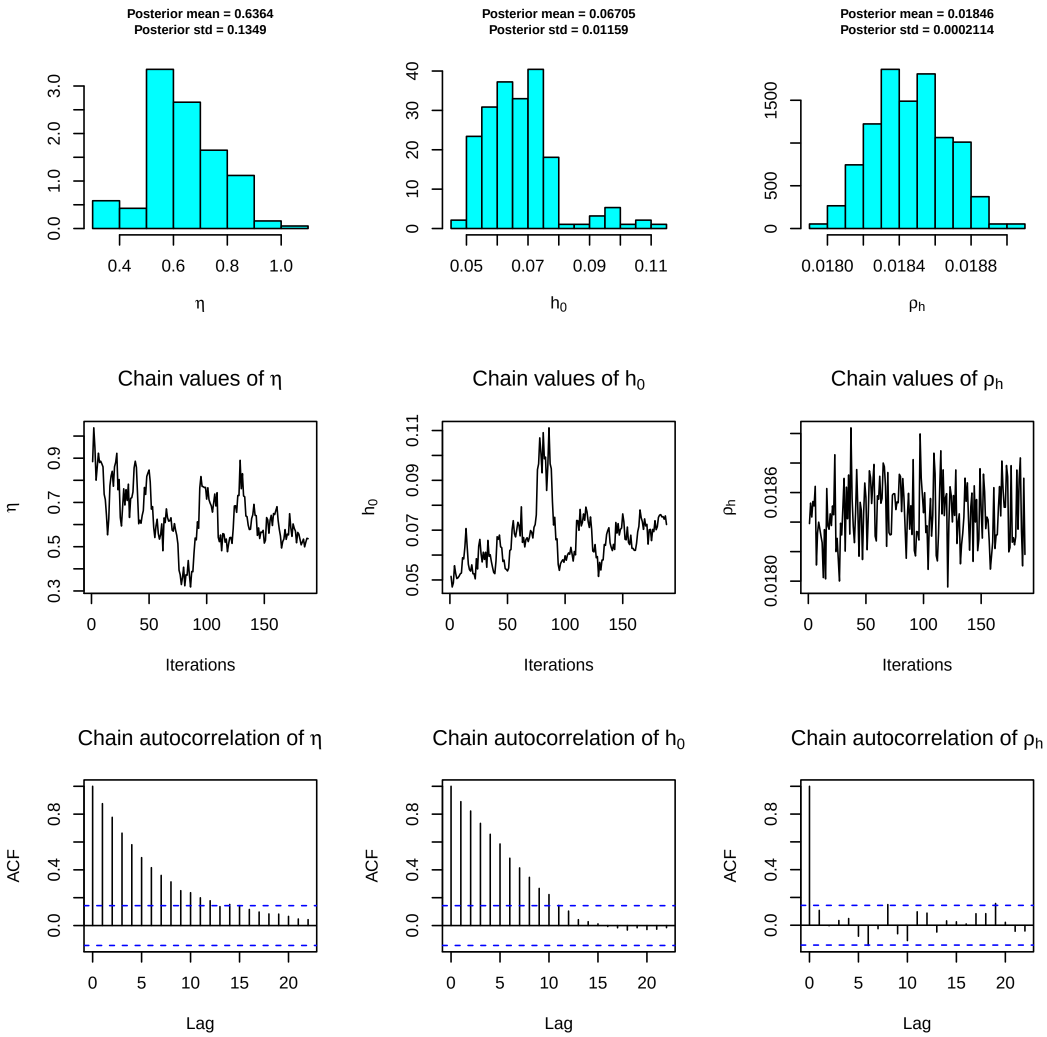

Figure 16: Posterior distribution, traceplots and autocorrelation plots of SEIQRHD model parameters η, h_0, and ρ_h.

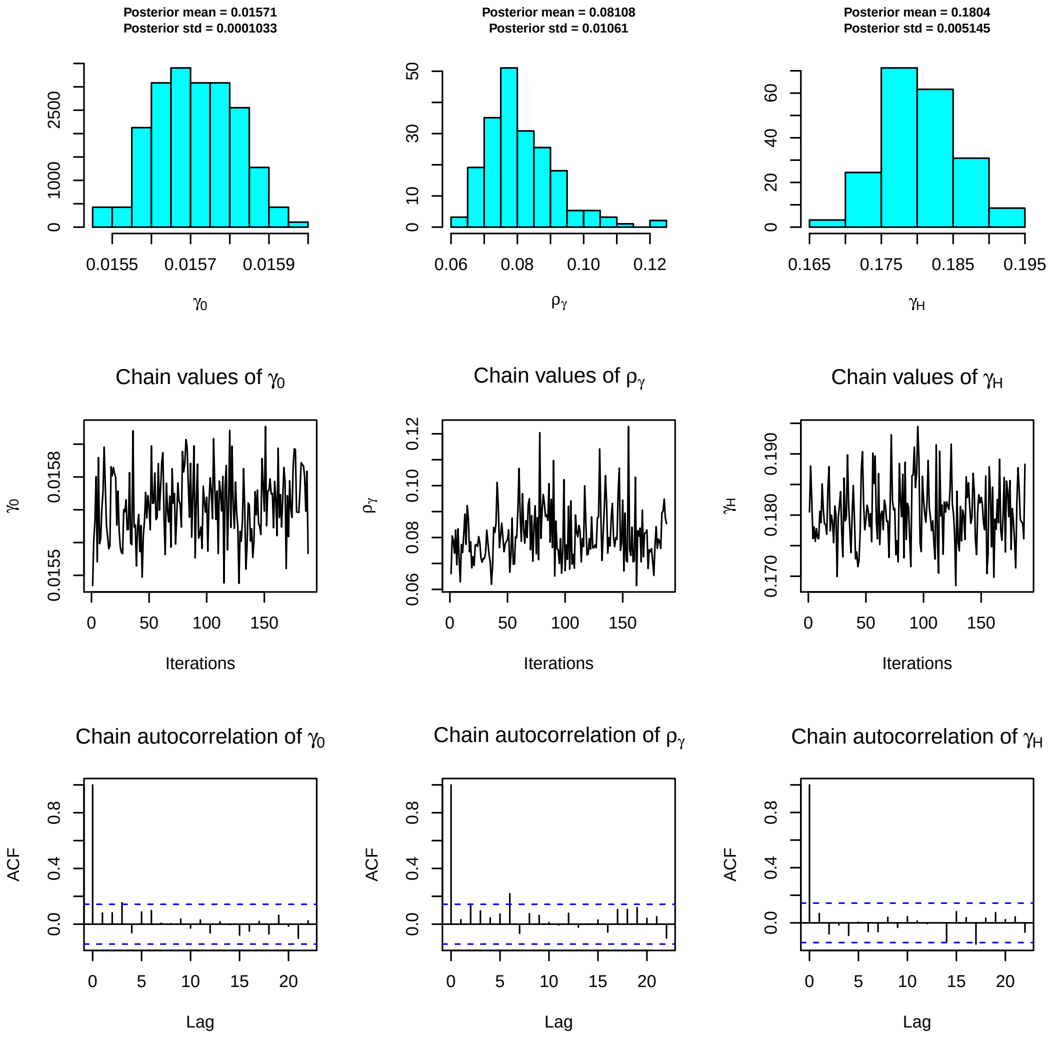

Figure 17: Posterior distribution, traceplots and autocorrelation plots of SEIQRHD model parameters γ_0, ρ_γ, and γ_H

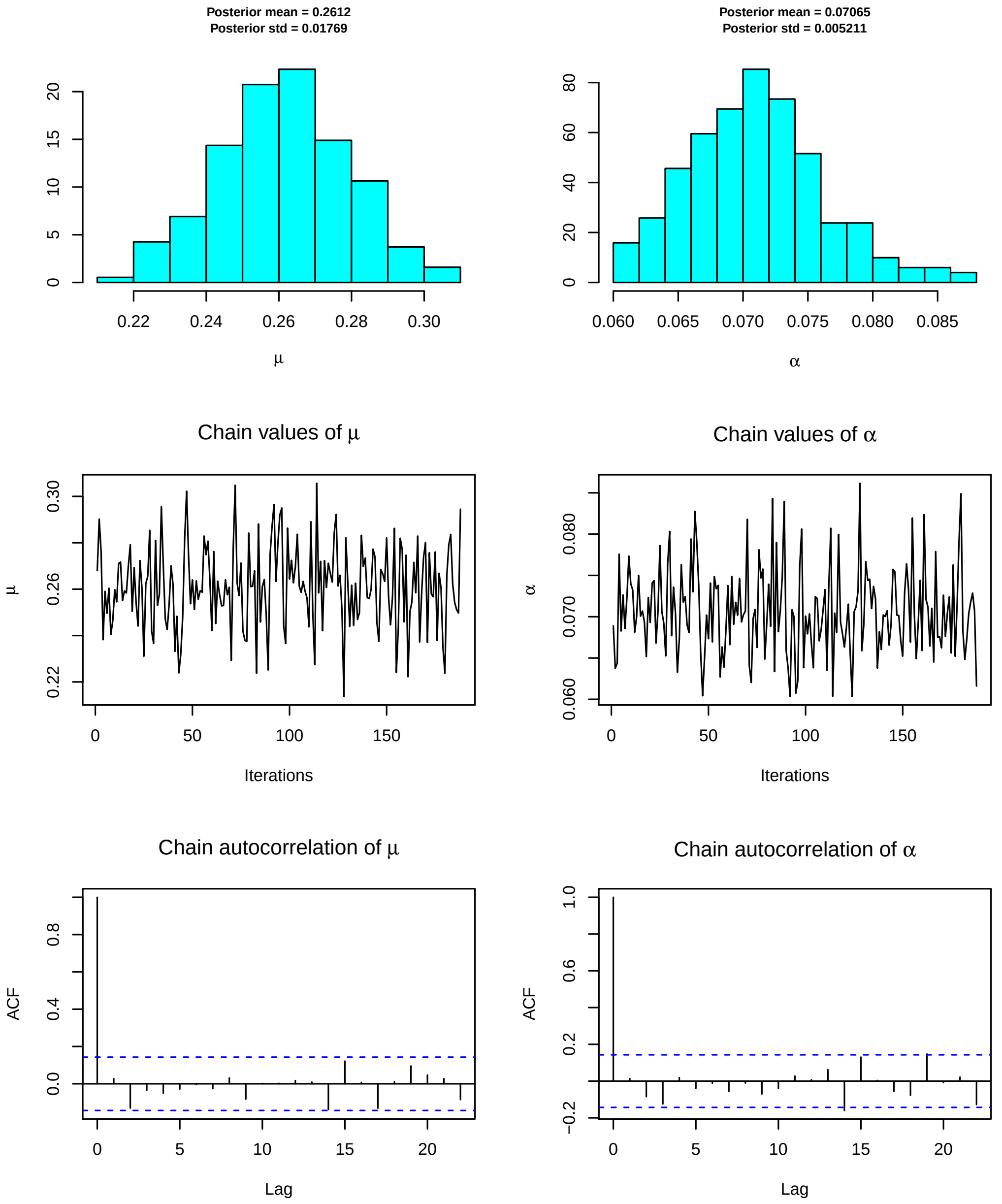

Figure 18: Posterior distribution, traceplots and autocorrelation plots of SEIQRHD model parameters μ and α
